# Surgical treatment for dropped head syndrome with cervical spondylotic amyotrophy: a case report

**DOI:** 10.1186/s13104-018-3612-2

**Published:** 2018-07-24

**Authors:** Shinji Taniguchi, Hiroshi Takahashi, Yasuchika Aoki, Arata Nakajima, Fumiaki Terajima, Masato Sonobe, Yorikazu Akatsu, Manabu Yamada, Takeo Furuya, Masao Koda, Masashi Yamazaki, Seiji Ohtori, Koichi Nakagawa

**Affiliations:** 1 Department of Orthopaedic Surgery, Toho University Sakura Medical Center, 564-1, Shimoshizu, Sakura City, Chiba 285-8741 Japan; 2 Department of Orthopaedic Surgery, Chiba Eastern Medical Center, 3-6-2, Okayamadai, Togane City, Chiba 283-8686 Japan; 3 Department of Orthopaedic Surgery, Chiba University Graduate School of Medicine, 1-8-1, Inohana, Chuoku, Chiba City, Chiba 260-8677 Japan; 4 Department of Orthopaedic Surgery, University of Tsukuba, 1-1-1, Tennodai, Tsukuba City, Ibaragi 305-8575 Japan

**Keywords:** Dropped head syndrome, Cervical spondylotic amyotrophy, Spinal instrumented surgery

## Abstract

**Background:**

Dropped head syndrome (DHS) is a flexion deformity of the neck that is caused by severe weakness of the neck extensor muscles. DHS occurs in combination with not only neuromuscular disorders, but also cervical spondylosis. However, there are few reports of DHS complicated by cervical spondylotic amyotrophy (CSA). Here we report a case of DHS with CSA in a patient who underwent surgical treatment.

**Case presentation:**

A 79-year-old man became aware of dropped head and gait disturbance in addition to the paralysis of his right upper extremity. At his initial visit, he had a severe chin-on-chest posture. Neurological examination revealed severe paralysis of deltoid, biceps, wrist extensor, finger flexor, extensor, and abductors, in addition to lower extremity spasticity. Nevertheless, sensory dysfunction was not observed. X-ray images showed severe kyphosis at the upper thoracic level. MRI and CT myelography findings revealed spinal canal stenosis at the level of C5–6 and C6 root compression of the right side. Motor neuron disease was excluded because of findings from electromyography. Therefore, we diagnosed this patient as having DHS with cervical spondylotic amyotrophy. A C2–Th5 posterior fusion with C3–C6 laminoplasty and C5–6 foraminotomy on the right side were performed. After surgery, the complaint of dropped head was improved significantly and bilaterally finger motion was improved slightly. His neck position was maintained at the final follow-up at about 1 year after surgery.

**Conclusions:**

Despite the limitation of short-term follow-up, favorable results for the DHS were maintained in the present case. Surgical treatment for similar cases may be a feasible option, but surgery does have some complications.

## Background

Dropped head syndrome (DHS) is a flexion deformity of the neck that is caused by severe weakness of the neck extensor muscles. As a result, the patient has a chin-on-chest deformity, interference with forward vision, and severe disturbance of activities of daily living. There are several reports about the etiology of DHS. According to those reports, DHS occurs in combination with neuromuscular disorders such as noninflammatory myopathy, motor neuron disease, and Parkinson’s disease [1–6]. By contrast, there are several reports of DHS in combination with cervical spondylotic disorders such as cervical spondylotic myelopathy or DHS after cervical laminoplasty [7]. However, there are few reports of DHS complicated by cervical spondylotic amyotrophy (CSA) [1] and, to our knowledge, there are no reports of surgical treatment for this condition. The detailed mechanism of DHS remains unclear. Here we report a case of DHS with CSA in a patient who underwent surgical treatment.

## Case presentation

**Fig. 1:**
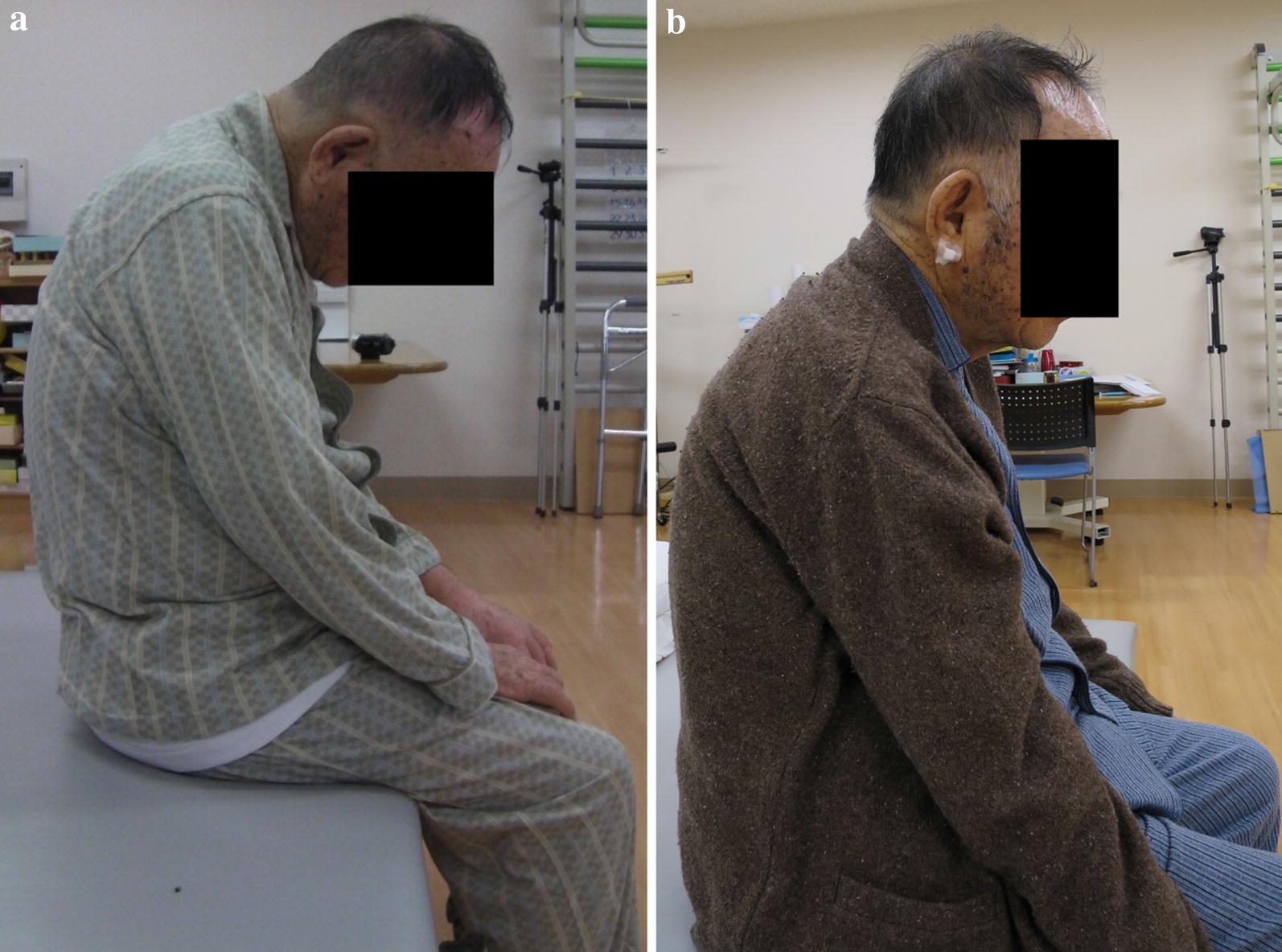
Photograph of the patient before surgery. **a** Before surgery. **b** At the final follow-up

**Fig. 2:**
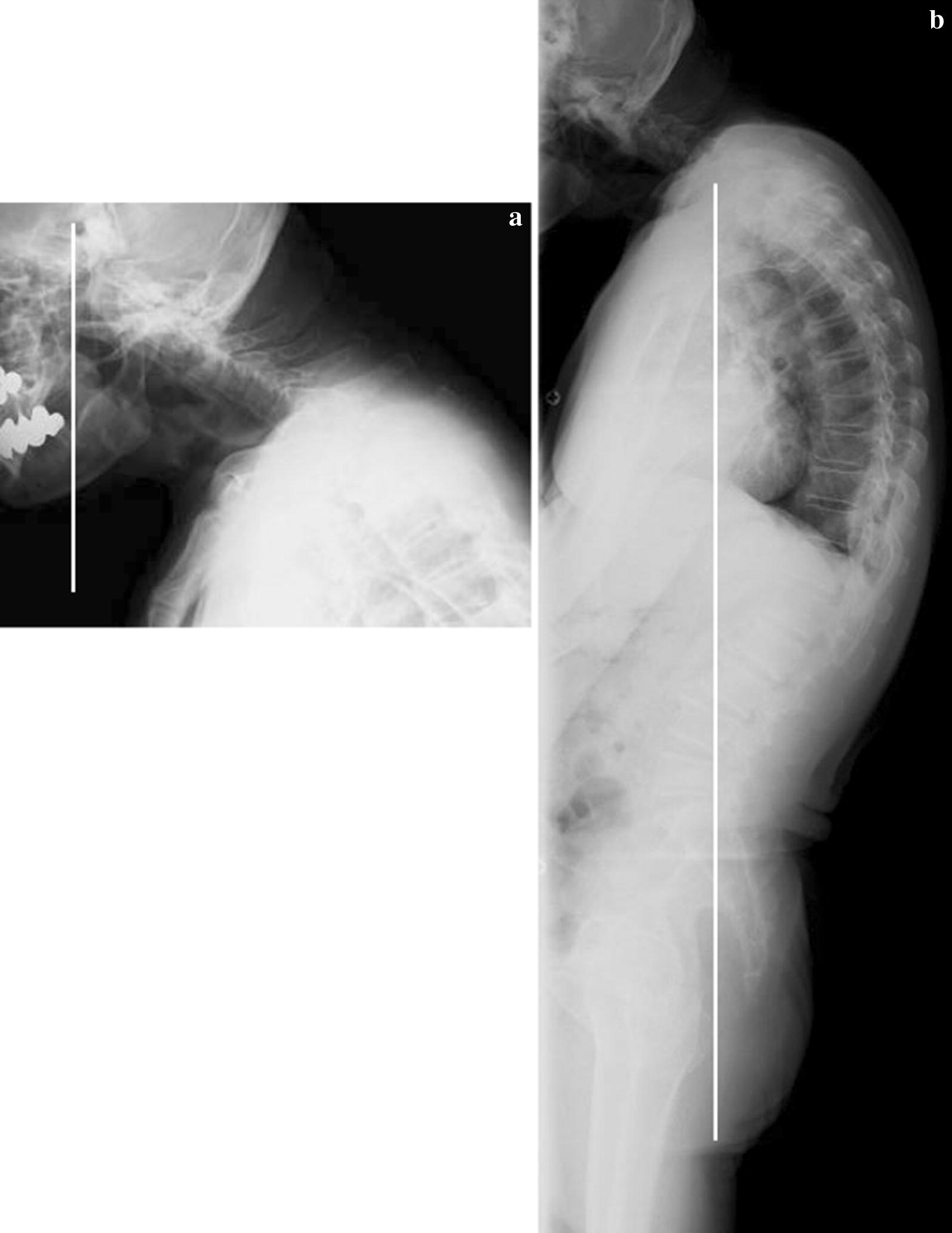
X-ray image before surgery. **a** Cervical X-ray lateral finding. White line is the center of gravity of the head-plumb line. **b** Whole spine X-ray lateral finding. White line is the C7-plumb line

**Fig. 3:**
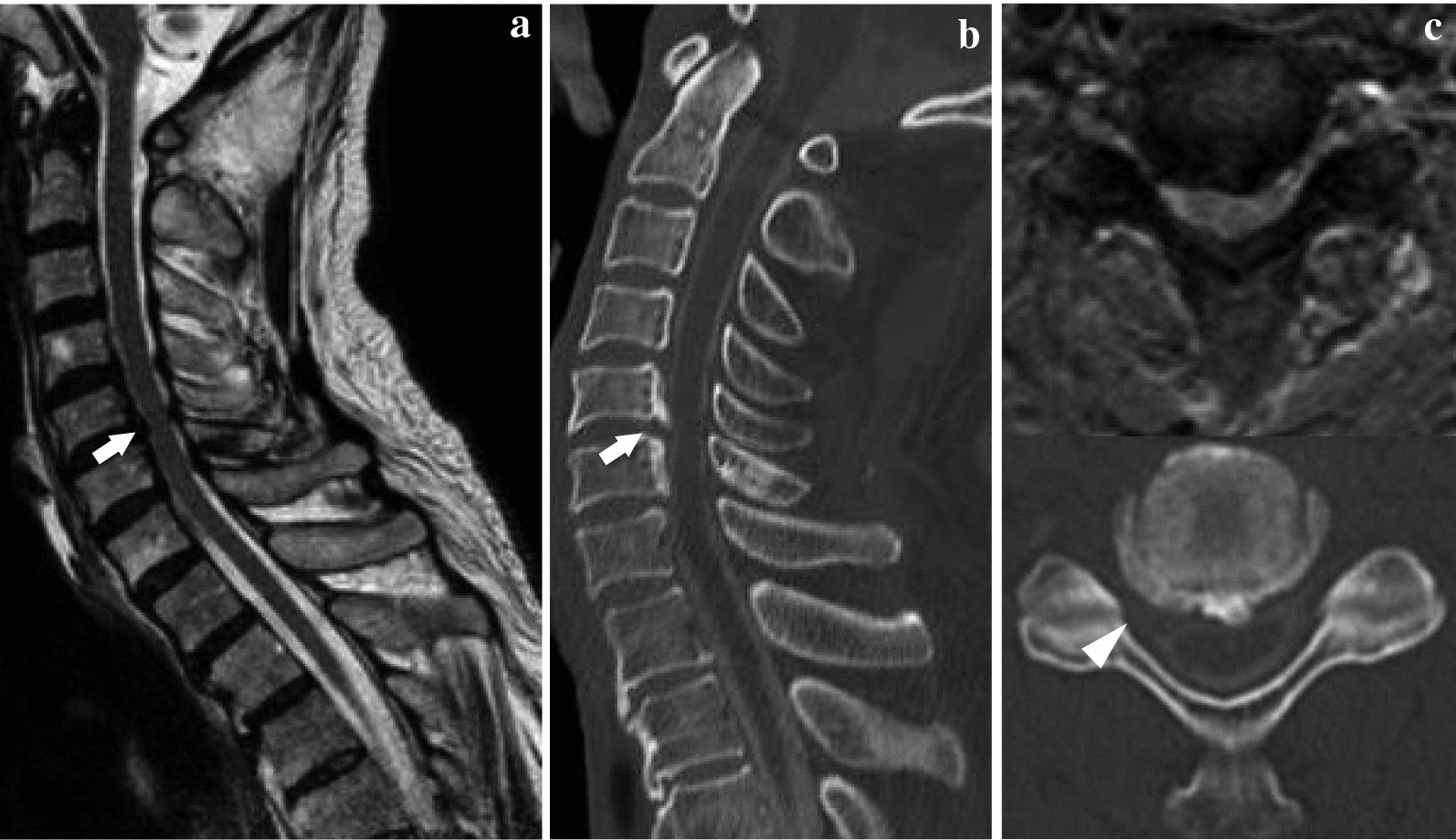
MRI and CT. **a** MRI T2-weighted imaging. Sagittal imaging showed spinal cord anterior indentation at the level of C5–6 (arrow). T2 high signal change was not observed. **b** CT myelography sagittal reconstruction imaging showed that the anterior indentation of spinal cord is because of ossification of the longitudinal ligament (arrow). **c** MRI T2-weighted imaging and CT myelography axial imaging at the level of C5–6 showing right side C6 nerve root compression (nerve root sleeve disappearance, arrow head) in addition to the spinal cord compression

**Fig. 4:**
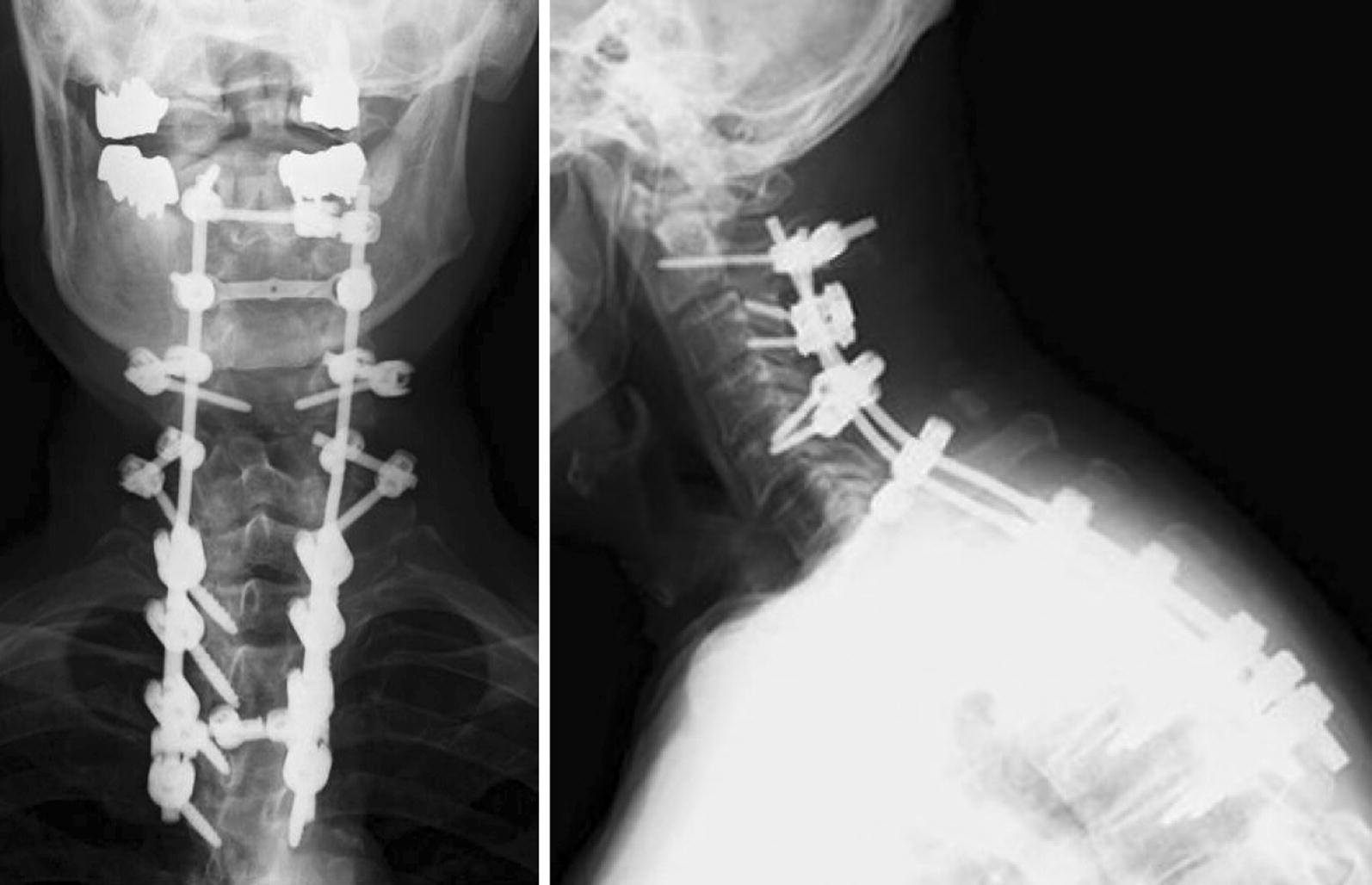
X-ray image at final follow-up

A 79-year-old man became aware of paralysis of his left fingers 2 years earlier. He was diagnosed as having cervical spondylotic amyotrophy and underwent a percutaneous endoscopic cervical posterior herniotomy at another hospital. However, after his surgery, his left finger became completely paralyzed. Furthermore, from 6 months after the initial surgery, he became aware of paralysis of his right upper extremity, gait disturbance, and dropped head. One month of conservative treatment using collar immobilization was used at the other hospital. Despite the treatment, his symptoms did not improve, and ultimately he presented to our hospital. He had a history of hypertension and diabetes. At his initial visit, he had a severe chin-on-chest posture (Fig. 1a). Neurological examination revealed severe paralysis of his right-side deltoid, biceps, wrist extensor, finger flexor (MMT grade 3), finger extensor (MMT grade 2), and abductors (MMT grade 1). By contrast, his left side upper extremity showed almost complete paralysis. The deep tendon reflex was increased at his lower extremity bilaterally, although it was absent at his upper extremity bilaterally. Because of sustained clonus of his ankle joint bilaterally, he had severe spasticity and could not walk unaided. However, sensory dysfunction was not observed. The Japanese Orthopaedic Association (JOA) score was 9.5 points. X-ray images showed severe kyphosis at the upper thoracic level. The center of gravity line from the head to C7 sagittal vertical axis (CGH-C7 SVA), which measured the deviation of the center of gravity of the head-plumb line (extending from the anterior margin or the external auditory canal) was 135 mm. The C2–C7 angle showed 2° lordosis. Otherwise, the C2–Th5 angle showed 38° kyphosis. Pelvic incidence was 44°, lumbar lordosis was 49°, and C7 sagittal vertical axis (C7-SVA) was 0 mm from the whole spine X-ray image. As the result, he had dropped head due to cervical and upper thoracic kyphosis and thoracolumbar sagittal balance was maintained (Fig. 2). MRI and CT myelography revealed spinal canal stenosis at the level of C5–6 because of ossification of the posterior longitudinal ligament of the spine and C6 root compression on the right side because of a foraminal osteophyte (Fig. 3). Although paraspinal muscle intensities were not observed in MRI, serum CK was increased to 584 (U/L). Electromyography showed chronic denervation at lower cervical level (C5–C8) and myogenic pattern at the paraspinal muscles was not observed that suspected the possibility of secondary myopathy. Parkinsonism was not also observed. Diabetes neuropathy, amyotrophic lateral sclerosis (ALS), myopathy, and Parkinson’s disease were excluded by the neurologist because of nerve conduction velocity and findings from electromyography. From the neurology, imaging, and electromyography findings, we diagnosed the pathogenesis of the DHS in this patient as follows: C5–6 anterior horn damage caused DHS as a consequence of back muscle atrophy at the C7 to upper thoracic levels and paralysis of the right finger; right side C6 anterior root damage caused paralysis of the deltoid and biceps; and the C5–6 pyramidal tract sign caused severe spasticity of the lower extremity bilaterally. Despite conservative treatment by collar immobilization, neither the DHS nor paralysis were improved. Therefore, surgical treatment was chosen. A C2–Th5 posterior fusion with C3–C6 laminoplasty and C5–6 foraminotomy on the right side were performed. Immediately after surgery, the complaint of dropped head was improved significantly and bilaterally finger motion was improved slightly. The neck position was maintained (Fig. 1b) and the patient could walk using a circular walker mobility aid at the final follow-up 1 year after surgery. The JOA score was improved up to 10.5 points and the recovery rate of JOA was 13%. At the final follow-up, X-ray imaging showed CGH-C7 SVA decreased to 50 mm and C2–Th5 angle increased to 4° lordosis (Fig. 4). Unfortunately, the patient died of pneumonia 2 months after the 1 year follow-up.

## Discussion and conclusions

Several reports have indicated favorable results of surgical treatment for DHS [8]. However, surgical treatment for DHS sometimes has complications for the adjusted segments [7–9]. Generally, DHS is classified as a flexible type in which the neck position can be corrected by passive head extension or a supine position, and a rigid type in which the neck position cannot be corrected by changing posture [10]. In the present case, the sagittal alignment was corrected at the supine position as seen by CT myelography. In addition, he had upper thoracic kyphosis complicated with cervical kyphosis. Therefore, we chose posterior decompression and fusion surgery and extended the distal fusion level to T5. If the sagittal alignment is not corrected at the supine position, indicating a rigid type, then anterior release or osteotomy in addition to the posterior decompression and fusion may be needed. The outcome was a favorable result for the dropped head and disturbance of forward vision that was maintained until the final follow-up.

By contrast, neurological improvement was slight in this case. In this case, his left finger became completely paralyzed after the surgery at previous hospital. However, the dropped head, right finger paralysis, and gait disturbance started after 6 months after the initial surgery. Considering the period and no improvement until the final follow-up, we speculate that the cause of his left finger paralysis is nerve root injury during the initial surgery at previous hospital and the initial posterior herniotomy does not concerns to the dropped head. A previous report that the surgical outcome for CSA is fair, especially in the distal type, is consistent with the outcome of this case [11]. The pathophysiology of CSA is segmental damage to the anterior horn or anterior root. Although CSA occurs secondary to cervical spondylosis, the severity of spondylotic change is not strictly correlated to the occurrence of CSA [11]. In addition, the T2 high signal change in cervical MRI is not constant. Thus, other neuromuscular disorders need to be excluded for a diagnosis of CSA. However, differentiating CSA from ALS is initially difficult [12]. There are some reports of DHS complicated with CSA [1, 12]. According to Ahdab et al., as distinct from our case, the dropped head occurred first, and shoulder muscle weakness occurred subsequently. Furthermore, the first diagnosis was ALS and the final diagnosis changed to CSA after 6 years [1]. In our present case, although the neurologist excluded a motor neuron disease such as ALS, the fair neurological outcome and the outcome of the patient may suggest that the diagnosis is CSA complicated with ALS. However, considering all the various factors together, the dropped head in this case can be potentially explained by secondary myopathy due to CSA. Nevertheless, the outcome for the patient was satisfactory with improvement of the dropped head and forward vision. Thus, surgical treatment for such a patient can be a feasible option despite the life prognosis, although it has some complications.

In conclusion, we experienced a case of DHS with CSA. Despite the limitations of the short-term follow-up, a favorable result was obtained for the dropped head and forward vision in the present case. Surgical treatment for similar cases may be a feasible option, although it may have some complications. Further experience of such cases and investigation is needed.

## Abbreviations

DHS: dropped head syndrome
CSA: cervical spondylotic amyotrophy
MMT: manual muscle test
JOA: Japanese Orthopaedic Association
CHG-C7 SVA: center of gravity line from the head-C7 sagittal vertical axis
C7-SVA: C7 sagittal vertical axis
ALS: amyotrophic lateral sclerosis
